# Intranasal recombinant protein subunit vaccine targeting TLR3 induces respiratory tract IgA and CD8 T cell responses and protects against respiratory virus infection

**DOI:** 10.1016/j.ebiom.2025.105615

**Published:** 2025-02-20

**Authors:** Katharina Wørzner, Signe Tandrup Schmidt, Julie Zimmermann, Ahmad Tami, Charlotta Polacek, Carlota Fernandez-Antunez, Katrine Top Hartmann, Rune Fledelius Jensen, Julia Sid Hansen, Kristin Illigen, Louise Krag Isling, Gitte Erbs, Gregers Jungersen, Ida Rosenkrands, Anna Offersgaard, Judith Gottwein, Kenn Holmbeck, Henrik Elvang Jensen, Santseharay Ramirez, Frank Follmann, Jens Bukh, Gabriel Kristian Pedersen

**Affiliations:** a Department of Infectious Disease Immunology, Center for Vaccine Research, Statens Serum Institut, Copenhagen, Denmark; b Virus Research & Development Laboratory, Department of Virology and Microbiological Special Diagnostics, Statens Serum Institut, Copenhagen, Denmark; c Copenhagen Hepatitis C Program (CO-HEP), Department of Immunology and Microbiology, Faculty of Health and Medical Sciences, University of Copenhagen, Copenhagen, Denmark; d Copenhagen Hepatitis C Program (CO-HEP), Department of Infectious Diseases, Copenhagen University Hospital, Hvidovre, Denmark; e Department of Veterinary and Animal Sciences, University of Copenhagen, Copenhagen, Denmark

**Keywords:** SARS-CoV-2, CAF®09b, CD8 T cells, Intranasal vaccine

## Abstract

**Background:**

Intranasal vaccines against respiratory viruses are desired due to ease of administration and potential to protect against virus infection of the upper respiratory tract.

**Methods:**

We tested a cationic liposomal adjuvant delivering the TLR3 agonist Poly (I:C) (CAF®09b) for intranasal administration, by formulating this with SARS-CoV-2 spike trimeric protein and assessing airway mucosal immune responses in mice. The vaccine was further evaluated in SARS-CoV-2 virus challenge models, using mice expressing the human ACE2 receptor and Syrian hamsters.

**Findings:**

The intranasal vaccine elicited both serum neutralising antibody responses and IgA responses in the upper respiratory tract. Uniquely, it also elicited high-magnitude CD4 and CD8 T cell responses in the lung parenchyma and nasal-associated lymphoid tissue. In contrast, parenteral administration of the same vaccine, or the mRNA-1273 (Spikevax®) vaccine, led to systemic antibody responses and vaccine-induced CD4 T cells were mainly found in circulation. The intranasal vaccine protected against homologous SARS-CoV-2 (Wuhan-Hu-1) challenge in K18-hACE2 mice, preventing weight loss and virus infection in the upper and lower airways. In Syrian hamsters, the vaccine prevented weight loss and significantly reduced virus load after challenge with the homologous strain and Omicron BA.5.

**Interpretation:**

This study demonstrates that intranasal subunit vaccines containing TLR3-stimulating cationic liposomes effectively induce airway IgA and T cell responses, which could be utilised in future viral pandemics.

**Funding:**

This work was primarily supported by the 10.13039/501100007601European Union Horizon 2020 research and innovation program under grant agreement no. 101003653.

**Research in context**

**Evidence before this study**

Intranasal vaccines containing an effective mucosal adjuvant may induce immunity in the upper respiratory tract with the potential to block respiratory pathogens at the portal of entry. In contrast, intramuscularly administered vaccines effectively protect against severe disease but are ineffective at eliciting immune responses in the upper airways.

**Added value of this study**

Our study demonstrates an intranasal protein subunit vaccine incorporating a TLR3 agonist, which effectively induced IgA and CD8 T cell responses in the upper airways and protected against a severe respiratory virus infection. In contrast, when the same vaccine or a state-of-the-art mRNA vaccine (mRNA-1273) was given intramuscularly it mainly elicited systemic immune responses.

**Implications of all the available evidence**

This study supports that mucosal vaccines using adjuvanted recombinant protein may effectively protect the upper airways against respiratory virus infection.

## Introduction

Licenced Severe Acute Respiratory Syndrome Coronavirus 2 (SARS-CoV-2) vaccines were highly effective against the first wave of COVID-19.^1^, ^2^, ^3^ However, immunity rapidly waned concurrent with the emergence of virus variants evading antibody responses. Furthermore, the first-generation SARS-CoV-2 vaccines elicited only limited immune responses in the upper airways and were thus ineffective at protecting against virus transmission. Parenteral vaccines predominantly induce systemic immune responses, but pandemic viruses, including SARS-CoV-2, are mainly transmitted by respiratory droplets and infect the upper respiratory tract, which is not effectively protected by circulating antibodies.^4^^,^^5^

Intranasal (i.n.) vaccination can induce secretory IgA at the nasal epithelium that may prevent initial viral replication. Licenced i.n. vaccines include the live attenuated influenza vaccine Flumist® and recently a number of adenoviral vectored vaccines have been developed for SARS-CoV-2.^6^^,^^7^ These were marketed late in the pandemic and their effectiveness as pre-exposure vaccines is unclear. The best correlate of protection for i.n. administered vaccines may be IgA responses in the upper airways rather than systemic neutralising antibody responses.^8^^,^^9^ Immunisation i.n. also elicits local tissue resident memory (T_RM_) CD4 and CD8 T cell responses in nasal-associated lymphoid tissue (NALT) and lungs.^10^^,^^11^ This is important because several studies have shown that cell-mediated immune (CMI) responses are long-lasting^12^^,^^13^ and therefore may be protective even when antibody responses have waned. Furthermore, T cells may recognise mutated viruses better than antibodies, which is particularly important in case of emerging virus variants.^14^^,^^15^ However, although there is substantial evidence that CMI responses can protect against respiratory viruses,^16^, ^17^, ^18^ clinical trials with vaccines inducing only CMI responses have been largely disappointing so far. For instance, Modified Vaccinia Ankara encoding influenza virus nucleoprotein (NP) and matrix-1 (M1) protein did not provide added benefit to standard vaccination.^19^ One possibility for the low efficacy could be that the vaccine, which was given parenterally, may not have elicited effective CMI responses in the airways. Thus, strategies to elicit airway immunity are desired, particularly vaccines inducing both antibody and cellular immune responses in the respiratory tract.

For SARS-CoV-2, parenteral immunisation with the mRNA vaccine BNT162b2 did not elicit airway CD8 T cell responses, but CD8 T cell responses were observed in the lungs upon subsequent i.n. unadjuvanted spike protein boosting.^20^ In contrast, the i.n. vaccine alone did not generate CD8 T cell responses in the lungs.^20^ In this study, we aimed to evaluate a novel liposomal adjuvant targeting TLR3 (CAF09b) for intranasal administration. When combined with the recombinant spike HexaPro protein^21^ and administered i.n., this adjuvant elicited both IgA and CD4 and CD8 T cell responses in the respiratory tract and protected against SARS-CoV-2 infection in the highly susceptible K18-hACE2 mouse model. In Syrian hamsters, the vaccine prevented weight loss and significantly reduced virus titres in the upper and lower airways. Overall, i.n. immunisation with protein subunit vaccines may be an effective strategy to elicit protective airway mucosal IgA and T cell responses.

## Methods

### Ethics

Animal studies were conducted in accordance with European Community Directive 2010/63/EU (current consolidated version 26/6-2019). The experiments were accepted by the National Animal Experiments Council under the licences 2017-15-0201-01363 and 2020-15-0201-00554.

### Antigens and adjuvants

Recombinant SARS-CoV-2 prefusion-stabilised spike ectodomain HexaPro trimer^21^ was produced by transient expression in freestyle 293-F cells as described previously.^22^^,^^23^ CAF®01 and CAF®04, containing dimethyldioctadecylammonium (DDA) and the MINCLE agonist trehalose dibehenate (TDB) or monomycoloyl glycerol (MMG), respectively, were produced by the thin film hydration method as described previously.^24^ CAF®09b, containing the same components as CAF04 with the addition of the TLR3 agonist Poly(I:C), was produced analogously. The adjuvants were used at doses of 250 μg DDA/50 μg MMG (CAF04) or 250 μg DDA/50 μg MMG/12.5 μg Poly (I:C) (CAF09b) in 10 mM TRIS buffer with 2.2% glycerol (pH 7.0). Adjuvant formulations were analysed visually for potential flocculation and characterised for particle size and polydispersity index (PDI) by dynamic light scattering, using the photon correlation spectroscopy technique. The surface charge of the particles was analysed by measuring the zeta potential (laser-Doppler electrophoresis). Measurements were performed at 25 °C by using a Zetasizer Nano ZS (Malvern Instruments, Worcestershire, UK) equipped with a 633 nm laser and 173° detection optics. Malvern DTS v.6.20 software was used for data acquisition and analysis. Particle size distribution was reflected in the PDI, which ranges from 0 for a monodisperse to 1.0 for a heterodisperse formulation.

### ELISA for spike-ACE2 binding

ACE2 binding ELISA was performed to assess if spike-ACE2 binding was intact after formulation in CAF09b. Maxisorp Plates (Nunc, Denmark) were coated overnight with recombinant human ACE2 (ACE-HM101, Kactus Biosystems) at 2 μg/mL in carbonate buffer pH 9.6. Plates were washed with PBS containing 0.2% Tween 20 and blocked with 2% skimmed-milk powder (SM). After blocking, spike HexaPro protein alone or formulated in CAF09 were added serially diluted in PBS with 1% SM, followed by incubation with hamster-anti-spike protein serum (generated in-house by immunising hamsters with HexaPro spike protein) as primary antibody. HRP-conjugated goat anti-hamster IgG antibody (Invitrogen, AB_2536572) was used as secondary antibody and TMB-PLUS (Kem-En-Tec, Denmark) as substrate, and the absorbance was recorded at 450 nm with subtraction of the absorbance value at 620 nm for background correction.

### Human TLR3 reporter cell line

Human TLR3 Reporter HEK293 Cells (NF-κB) were obtained from Invivogen. The cell line has been validated using Poly (I:C) as positive control. Secreted embryonic alkaline phosphatase levels produced upon TLR3 stimulation were determined by performing the assay in HEK-Blue™ Detection medium. Cells were stimulated with Poly(I:C) alone (naked) or formulated in CAF09b or CAF04 (same components as CAF09b but without Poly(I:C)) (negative control). The concentrations were optimised for each formulation and were 100, 20, 4, and 0.8 μg/mL for naked Poly(I:C) and 1.67, 0.42, 0.1, 0.026, and 0.0065 μg/mL for Poly(I:C) in CAF09b. The absorbance was measured at 655 nm 20 h post stimulation (Molecular Devices SpectraMax iD3 Multi-Mode Microplate Reader).

### Animals

Female C57Bl/6 (C57BL/6JOlaHsd) wild type mice, 7–9 weeks old, were obtained from Envigo (The Netherlands). K18-hACE2 mice (B6; C3-Tg(CAG-ACE2)70Ctkt female), 7–9 weeks old, were ordered from Taconic. Male Syrian hamsters (*Mesocricetus auratus*), nine weeks old, were obtained from Janvier. Animals were housed in the animal facilities at Statens Serum Institut and maintained in rooms with controlled environment (20–23 °C; relative humidity 55 ± 10%; 12/12 h light/dark cycle). The mice were randomly allocated to conventional or IVC cages (type III polycarbonate cages (820 cm^2^)) with up to eight mice per cage. The hamsters were housed in polycarbonate cages type IV (1820 cm^2^) with high lid (in total app. 30 cm. high) with up to four animals/cage. All animals were given Aspen bedding and bricks (Tapvei), EnviroDri (LBS) and tunnels or houses made of polycarbonate. Mice were also offered DesRes paper houses (LBS) while hamsters had hay-bricks (Vangs) and twisted paper rolls (“Diamond Twist” Envigo Teclad) hanging in their cage lids. Irradiated sunflower seeds, corn grains and peanuts or bits of carrots were given to animals once a week. Pelleted diet (Envigo Teclad 2916) and tap water was provided ad libitum.

### Immunisations

Mice and hamsters were administered with two immunisations 14 or 21 days apart (as stated in figure legends), using 5 μg/dose (mice) or 20 μg/dose (hamsters) of recombinant SARS-CoV-2 prefusion-stabilised HexaPro spike ectodomain formulated in CAF®01, CAF®04 or CAF®09b. The vaccines were either given as two subcutaneous (s.c.) immunisations (s.c./s.c.), as s.c. immunisation followed by intranasal (i.n.) boost (s.c./i.n.) or as two (i.n./i.n.) or three (i.n./i.n./i.n.) intranasal administrations. The s.c. immunisations were given at the base of tail (mice) or in the scruff of the neck (hamsters) at a final volume of 200 μL per immunisation. The i.n. immunisations were performed under isoflurane anaesthesia. Mice were given a volume of 10 μL per nostril. The mRNA-1273 (Spikevax®) vaccine was administered intramuscularly (i.m.) in 50 μL volume, corresponding to a 1 μg mRNA dose. Hamsters were placed in supine position and given 15 μL of vaccine pr. nostril. The number of animals in each group is stated in the figure legends.

### Sampling and organ preparation

Animals were euthanised by CO_2_ (80%)/O_2_ (20%), after anaesthetization with Zoletil-mix (Zolazepam, Tiletamin, Xylazin, and Butorphanol). Where indicated, mice were anesthetised with Zoletil-mix and then injected i.v. with 2.5 μg/250 μL anti-CD45.2-FITC (104, BD 553772) before euthanization (3–6 min later). Organs from mice were collected in RPMI-1640. NALT was isolated by placing the mouse on its back and removing the lower jaw to expose the palate. A small transvers incision was made right behind the incisor teeth and in the soft palate just behind the last molar. The palate was then the carefully removed/peeled off with a small forceps. NALT, spleens and lymph nodes were filtered through a 70 μm nylon mesh (BD Biosciences), washed and re-suspended in cell culture medium (RPMI-1640 supplemented with 2-mercaptoethanol, 1% pyruvate, 1% HEPES, 1% (v/v) premixed penicillin-streptomycin solution (Invitrogen Life Technologies), 1 mM glutamine, and 10% (v/v) foetal calf serum (FBS)). The nasal cavity was dissociated in 2.5 mL collagenase (Type II, 1 mg/mL, C6885-500 MG and type IV, 1 mg/mL, C5138-1G) and 15 μg/mL DNase I (Roche 10104159001) at 37 °C for 30 min under continuous shaking followed by filtration, centrifugation and resuspension in 1 mL PBS. Lymphocytes were isolated using Lympholyte® (Cedarlane CL5120) and harvested after 20 min of centrifugation. The cells were washed and re-suspended in cell culture medium. Lung tissue was minced and dissociated in C-tubes (Miltenyi Biotec) with 2.5 mL collagenase (Type IV, 1.6 mg/lung, C5138-1G) and DNase I (15 μg/mL, Roche 10104159001) using gentleMACS™ Dissociator (Miltenyi Biotec). Dissociated tissues were incubated for 1 h at 37 °C and then filtered through a 70 μm nylon mesh, washed and resuspended in cell culture medium.

### ELISA for antibody responses

Maxisorb Plates (Nunc) were coated overnight with 0.05 μg/well SARS-CoV-2 trimer (HexaPro) (4 °C). For antigen-specific IgG, plates were blocked with 2% BSA and serum was added diluted in PBS with 1% BSA. Polyclonal HRP-conjugated secondary rabbit anti-mouse IgG (Thermofisher, RRID: AB_138451) or goat anti-hamster IgG antibody (Invitrogen, AB_2536572), was diluted in PBS with 1% BSA. Nasal washes for antibody responses were sampled by flushing with 350 μL PBS +0.05% BSA. To measure antigen-specific antibodies in nasal washes of hamster, a rabbit anti-hamster HRP-coupled IgG/IgA/IgM antibody was used (Brookwood biomedical, sab3003). For mouse antigen-specific IgA (monomeric or dimeric), plates were blocked with 2% SM, and serum and nasal washes were added in PBS with 1% SM. Biotin-coupled goat anti-mouse IgA (Southern Biotech, AB_2794374) diluted in 1% SM was used as primary antibody, followed by streptavidin-HRP (BD, AB_2868972) diluted in PBS containing 0.2% Tween 20. Spike-specific antibodies were detected using TMB substrate (Kem-En-Tec Diagnostics), and the reaction was stopped with 0.5 M H_2_SO_4_. The absorbance was measured at 450 nm with subtraction of the absorbance value measured at 620 nm.

### Detection of spike antigen in nasal cavity

A sensitive chemiluminescent assay, S-PLEX SARS-CoV-2 Spike Kit (MSD), was used according to manufacturer's instructions to detect spike HexaPro protein in the nasal cavity. The plates were analysed on a Sector Imager 2400 system (Meso Scale Discovery).

### Neutralisation assay

Neutralisation of SARS-CoV-2 was performed using the SARS-CoV-2/human/Denmark/DK-AHH1/2020 isolate (GenBank accession number: MZ049597) cultured in Vero E6 cells, as previously described.^25^^,^^26^ This isolate is closely related to the Wu-Hu-1 sequence (with E309K and D614G substitutions in the spike protein). Cross-neutralisation studies were performed using the BA.5 omicron-variant SARS-CoV-2/human/DNK/DK-AHH6/2022 isolate (GenBank accession number: OP722492)^27^ cultured in Vero E6 cells. In brief, SARS-CoV-2 at a multiplicity of infection (MOI) of 0.01–0.15 was incubated 1 h at room temperature with serially diluted heat inactivated (at 56 °C for 30 min) plasma (1/100–1/51,200 dilution) at a 1:1 ratio. Following incubation, plasma/virus mixtures were added to naïve Vero E6 cells in quadruplicates in 96-well plates. After incubation for 48 h at 37 °C and 5% CO_2_, cells were fixed and stained as described.^25^ Single spots representing virus infected cells were counted by an Immunospot series 5 UV analyser, and percent neutralisation calculated. Neutralisation curves were constructed and the ID50 of plasma was calculated using non-linear regression (Log [inhibitor] vs normalised response [variable slope]), using GraphPad Prism. Four replicates of a 1/800 dilution (1.25 μg/mL) of a mouse derived SARS-CoV-2 spike neutralising antibody (Sino Biological #40592-MM57, RRID: AB_2857935) was used as positive control for each plate.^28^

### Cytokine profiling

The Mouse U-plex (IFN-γ, IL-17, IL-5, IL-13, and IL-10) assay was performed according to the manufacturer's instructions (Meso Scale Discovery) to measure CD4 T cell profiles after ex vivo re-stimulation of splenocytes with SARS-CoV-2 HexaPro trimer antigen (2 μg/mL cell culture medium incubated for 72 h at 37 °C and 5% CO_2_). The plates were analysed on a Sector Imager 2400 system (Meso Scale Discovery) and calculation of cytokine concentrations was performed by 4-parameter logistic non-linear regression analysis of the standard curve.

### Flow cytometry

One million cells were treated with Fc-block (BD Biosciences) and stained in PBS +1% FBS with cocktails of antibodies against the following surface proteins: CD45.2 FITC (104, BD 553772), CD4 APC-Cy7 (RM4-5, 1:600, eBioscience 47-0042-82), CD4 BV786 (GK1.5, 1:200, BD 563331), CD8a PerCP-Cy5.5 (53-6.7, 1:600, eBioscience 45-0081-82), CD8a BV421 (53-6.7, 1:600, Biolegend 100738), CD44 APC (IM7, 1:600, BD 559250), CD44 APC-Cy7 (IM7, 1:200, eBioscience 47-0441-82), CD62L PerCP-Cy5.5 (MEL-14, 1:200, BD 560513), CD19 PE-Cy7 (1D3, 1:600, BD 552854), CD11c PE-Cy7 (HL3, 1:200, BD 558079), and I-A/I-E BV605 (M5/114.15.2, 1:200, BD 563413). The Spike_539–546_ (VNFNFNGL)-specific MHC-I tetramer was obtained from NIH tetramer core facility. The MHC class II spike tetramer Spike_62–76_ (VTWFHAIHVSGTNGT)^20^ was custom made, using the corresponding peptide (JPT) and provided from the NIH tetramer core facility. A live/dead marker was used to exclude dead cells in the tetramer staining panels (Fixable Viability Dye eFluor™ 780, 1:500, eBioscience 65-0865-18 or eFluor 506, 1:500, eBioscience 65-0866-18). OVA-AF647 (Invitrogen O34784) was used to investigate retained antigen in the nasal cavity. For intracellular staining, antigen-re-stimulated cells (1 × 10^6^ cells/well) were initially stained with antibodies against surface proteins CD44 BV421 (IM7, 1:600, BD 563970), CD8a PerCP-Cy5.5 (53-6.7, 1:600, eBioscience 45-0081-82), and CD4 APC-eFluor780 (RM4-5, 1:600, eBioscience 47-0042-82). Following washes in PBS +1% FBS, the cells were fixed and permeabilized using Fix/Perm solution (BD Biosciences) followed by washes with Perm Wash solution (BD Biosciences). Cocktails of antibodies against intracellular cytokines (IFN-γ PE-Cy7 (XMG1.2, 1:200, eBioscience 25-7311-82), TNF-α PE (MP6-XT22, 1:200, eBioscience 12-7321-82), and IL-2 APC (JES6-5H4, 1:200, eBioscience 17-7021-82)) were added and cells were analysed on a BD Fortessa or FACSCanto flow cytometer. Data were analysed in FlowJo v. 10.1.

### SARS-CoV-2 challenge

SARS-CoV-2 challenge studies were performed in the BSL-III facilities at Statens Serum Institut. K18-hACE2-mice and hamsters were anaesthetised with isoflurane and inoculated i.n. with 3.1 × 10^3^ TCID_50_ in 50 μL per nostril of the Danish SARS-CoV-2 isolate SARS-CoV-2/human/DNK/SSI-H5/2020 (GenBank accession number: ON809567.1). This isolate is closely related to the Wu-Hu-1 sequence (with V367F and E990A substitutions in the spike protein and a G251V substitution in ORF3a). Alternatively, challenge was performed with the Omicron BA.5 isolate SARS-CoV-2/Hu/DK/SSI-H102/2023 (GenBank accession number: OY747658.1). Weight and signs of disease (lethargy, ruffled fur, hunched posture) were monitored until the study was terminated at five days post infection.

### RT-qPCR for SARS-CoV-2 detection

Lungs were dissociated by GentleMACS (M-tubes, Miltenyi). Nasal washes were sampled by flushing with 350 μL of PBS. RNA extractions were performed with the MagNA Pure 96 system (Roche Molecular Biochemicals, Indianapolis, Indiana, United States (US)), using MagNA Pure LC DNA Isolation kit I lysis buffer. All oligonucleotides were synthesised by Eurofins Genomics. RT-qPCR was performed using 5 μL of resuspended RNA in a 25 μL reaction volume using the Luna® Universal Probe One-Step RT-qPCR with Luna WarmStart® RT Enzyme Mix (New England Biolabs) with 400 nM concentrations primers and 200 nM of probe. Primers and probes for the SARS-CoV-2 E gene (diagnostic PCR) were as previously described.^29^ This method does not indicate if detected virus is actively replicating. Cycling conditions: 55 °C for 10 min, denaturation at 95 °C for 3 min, and 45 cycles of 95 °C (15 s) and 58 °C (30 s). A standard curve was generated using synthetic SARS-CoV-2 RNA ctr1 (MT007544.1) (Twist Bioscience). A stabilised RNA for 2019-nCoV E gene (EVAg) was used as positive control. Reactions were carried out using a Lightcycler-480 Real-Time PCR System (Roche). Results were expressed as log10-transformed numbers of genome equivalent copies per mL (nasal washes) or gram (lungs).

### Lung pathology in Syrian hamsters

The right lung lobes were pseudo-perfused fixed in 10% neutral buffered formalin and then placed in histo-cassettes for immersion fixation in 10% neutral buffered formalin for 24 h before being transferred to 70% ethanol. Paraffin embedded tissues were sectioned (4 μm) and stained with haematoxylin and eosin (H&E). Sections were evaluated for infiltration of inflammatory cells as previously described.^28^^,^^30^ Mononuclear cells were round to oval in shape and varied from 8 to 16 μm in diameter, they contained a single often round nucleus that varied in density and occupied from 30 to 90% of the cell having light blue stained cytoplasm. Neutrophils showed lobulated nuclei and neutrophilic cytoplasmic granules and varied between 10 and 12 μm in diameter. Type II hyperplasia was identified from the lining of the alveoli by cubic shaped epithelial cells. Syncytial cells were identified as large (up to 60–80 μm) densely stained multinucleated cells. In all sections the inflammatory reaction was scored as 0: absent and 1: present. All H&E stained lung sections were also scanned to digital slides and evaluated using Qupath software (version 0.4.2). For each animal, the area of the lung lobes was outlined and measured (μm^2^) using the wand brush annotation tool. Lung tissue affected by inflammation was outlined and measured (μm^2^), and the percentage of the total lung area affected by inflammation was calculated as the inflammation score.^31^ The investigators performing the histological examination were blinded to the experimental groups.

### Immunohistochemistry for SARS-CoV-2 nucleocapsid

Tissue sections of 4 μm were dewaxed and rehydrated through xylene and graded alcohol, respectively, quenched for endogenous peroxidase with 3% hydrogen peroxide in methanol for 15 min at room temperature. Following rinsing in TBS (50 mM Tris, 150 mM NaCl, pH 7.6), 2 × 5 min, sections were treated with Protease XXIV (0.018 g/100 mL) in TBS for 5 min for unmasking epitopes followed by rinsing in ice-cold TBS, 2 × 5 min, and then placed in Shandon Racks and blocked with Ultra V Block (Epredia, TL-125-HLJ) for 5 min. Slides were incubated with the primary antibody (Biosite 40143-T62, targeting SARS-2-CoV-2 Nucleocapsid) diluted 1:4000 in 1% BSA in TBS, pH 7.6 at 4 °C overnight followed by incubation with OneVision HRP Polymer (Epredia, TL-125-HLJ) for 30 min. Then they were incubated with AEC Vector (AEC substrate kit, Vector SK-4200) for 10 min. Tris-buffered saline with 0.1% Tween®20, pH7.6 was used for washing between the different incubations after adding the primary antibody. Subsequently, sections were counterstained in Mayer's haematoxylin (VWR, AMPQ00254.5000), and glass coverslips were mounted in glycerol-gelatin.

### Statistical analysis

Differences between groups were analysed by one-way ANOVA, using the CAF09 b s.c./s.c. group as reference and Dunnet's correction for multiple comparisons. Differences between mRNA-1273 (Spikevax) immunised and CAF09b immunised mice and between naïve and i.n./i.n. immunised groups in the SARS-CoV-2 infection model were analysed by a two-tailed unpaired t test (GraphPad v8.2.1). A survival curve was used to depict protection against virus challenge in hACE2 mice.

### Role of the funding source

The funding source had no role in study design, collection, analysis or interpretation of data or in the writing of the publication.

## Results

### Intranasal immunisation with CAF09b adjuvanted SARS-CoV-2 spike trimer induces upper airway IgA and CD8 T cell responses in mice

**Fig. 1:**
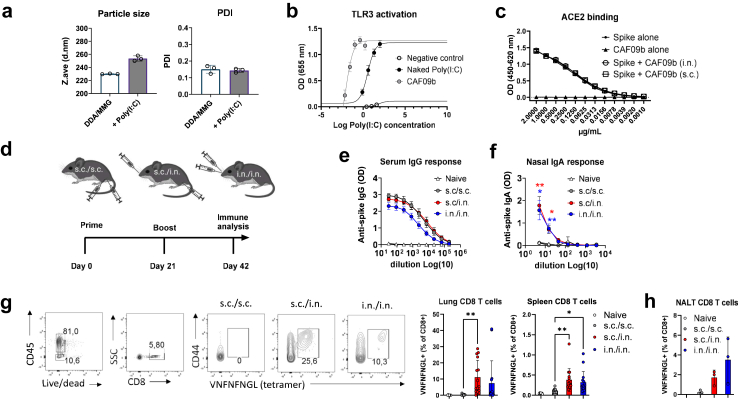
**Intranasal administration of CAF09b adjuvanted spike protein elicits IgA responses and antigen-specific CD8 T cells in the upper respiratory tract**. **a)** Poly (I:C) was formulated in DDA/MMG liposomes at a concentration of 12,500/2500/625 μg/mL DDA/MMG/Poly (I:C). This corresponds to a final dose of 250 μg/50 μg/12.5 μg DDA/MMG/Poly(I:C) for intranasal (i.n.) administration. The particle size (Z.ave) and polydispersity index (PDI) was measured using differential light scattering. **b)** A human TLR3 reporter (HEK293) cell line was used to assess delivery of Poly (I:C) in CAF09b. Cells were stimulated with Poly(I:C) alone (naked) or formulated in CAF09b at the indicated concentrations (100, 20, 4, and 0.8 μg/mL for naked Poly(I:C) and 1.67, 0.42, 0.1, 0.026, and 0.0065 μg/mL for Poly(I:C) in CAF09b). CAF04 (DDA/MMG without Poly(I:C)) was used as negative control. Secreted embryonic alkaline phosphatase produced upon TLR3 stimulation were determined using HEK-Blue™ Detection medium, measured as optical density (OD). **c)** The SARS-CoV-2 Spike HexaPro trimer was formulated in CAF09b adjuvant and confirmed to retain binding to ACE2 using ELISA. **d)** Mice were either left unvaccinated (naïve) or immunised with two doses of SARS-CoV-2 spike HexaPro trimer protein formulated in cationic liposomes (CAF®09b). The vaccine was administered as a conventional subcutaneous two dose regimen (s.c./s.c.), as subcutaneous priming followed by intranasal boosting (s.c./i.n.) or as two intranasal administrations (i.n./i.n.). Serum was sampled at 21 days after the 2nd immunisation. Created with BioRender.com. **e)** IgG antibody responses against the SARS-CoV-2 spike protein. **f)** Nasal wash IgA antibody responses against SARS-CoV-2 spike protein. Data are representative of n = 6 mice per group. Similar findings were obtained in another separate experiment (**e and f**). Spike-specific CD8 T cell responses were measured by flow cytometry. Mice were injected i.v. with anti-CD45.2 to distinguish between circulating and tissue resident cells. **g)** CD8 T cell responses in lungs and spleen. Cells were gated as CD45− (parenchymal cells not stained by the i.v., injected anti-CD45 antibody), CD8+ (to identify CD8 T cells), CD44+ (activation marker) SARS-CoV-2 spike-specific, using a tetramer (VNFNFNGL). Left panel shows representative stainings of lung cells. Data were pooled from two experiments with n = 6 mice and n = 9 mice, respectively. **h)** CD8 T cell responses in nasal-associated lymphoid tissue. The experiment was performed once and contained n = 9 mice. To obtain enough cells for analysis, each NALT data point was a pool from three mice. Mean ± SEM is displayed. Statistically significant differences are indicated by ∗ or ∗∗ (one-way ANOVA, comparing the mean of each column with the mean of the s.c./s.c. group, p < 0.05 or 0.01, respectively). There were no statistically significant differences among groups unless indicated.

The adjuvant CAF09b, composed of dimethyldioctadecylammonium (DDA), the MINCLE agonist monomycoloyl glycerol (MMG) and the TLR3 agonist Poly(I:C), is effective at inducing CD8 T cell responses and is currently tested in neoepitope peptide-based vaccines against metastatic melanoma in humans.^32^^,^^33^ Based on previous pre-clinical studies using other DDA-based adjuvants for i.n. administration,^28^^,^^34^ we hypothesised that the CAF09b adjuvant administered i.n. would be capable of eliciting IgA responses in the airways and that the addition of Poly(I:C) could aid in eliciting airway CD8 T cells. As proof-of-concept, we used SARS-CoV-2 HexaPro spike protein formulated in CAF09b as a model vaccine. We formulated a concentrated version of CAF09b corresponding to a full murine dose of 250 μg/50 μg/12.5 μg DDA/MMG/Poly(I:C) for i.n. administration. Compared to DDA/MMG alone, the particle size increased from 230 nm to 280 nm, when Poly(I:C) was incorporated, whilst both formulations were homogenous (Fig. 1a). We then confirmed CAF09b as an effective TLR3 stimulating delivery system in human HEK293 hTLR3 reporter cells, where poly(I:C) formulated in DDA/MMG (CAF09b) liposomes activated TLR3 at concentrations 400 times lower than its naked poly(I:C) counterpart (8.8 ng/mL and 3.5 μg/mL for CAF09b and naked poly(I:C), respectively) (Fig. 1b). Spike antigen was intact and could still bind to the ACE2 receptor after being formulated in CAF09b, both in standard concentrations for s.c. administration and when concentrated for i.n. delivery (Fig. 1c).

To evaluate CAF09b for i.n. administration, CAF09b-adjuvanted spike was administered to C57BL/6 mice either by subcutaneous only immunisation (s.c./s.c.), by subcutaneous prime followed by intranasal boost (s.c./i.n.), or as a two-dose i.n. only regimen (i.n./i.n.) (Fig. 1d). s.c./i.n. immunisation elicited similar systemic anti-spike IgG responses as s.c./s.c. immunisation (Fig. 1e). i.n./i.n. immunisation gave slightly lower systemic IgG responses, but IgA responses in nasal washes were similar as for the s.c./i.n. regimen. In contrast, s.c./s.c. immunisation did not elicit detectable IgA responses in nasal washes (Fig. 1f).

To investigate if i.n. immunisation with spike HexaPro formulated in CAF09b could elicit respiratory tract CD8 T cell responses, we stained CD8 T cells with a Spike_539–546_ (VNFNFNGL)-specific MHC-I tetramer.^20^ We distinguished between vascular (IV+) and parenchymal cells (IV−) by injecting anti-CD45 antibody before euthanasia.^35^ Mice that had received CAF09b-adjuvanted vaccine s.c./i.n. or i.n./i.n. had spike-specific CD8 T cells amongst activated (CD44+) T cells in the lung and spleen parenchyma (IV−) (Fig. 1g). Similarly, s.c./i.n. and i.n./i.n. administration of CAF09b adjuvanted vaccine elicited spike-specific CD8 T cells in the NALT (IV−), whilst these could not be detected after s.c./s.c. administration (Fig. 1h). CD8 T cell induction was at least partially dependent on the TLR3 agonist Poly(I:C), as i.n./i.n. immunisation with spike formulated in CAF04 (DDA/MMG) elicited lower CD8 T cell responses in NALT (not significant, p = 0.05), nasal cavity (significant, p < 0.05) and spleen (p = 0.07 and p < 0.01 in experiment #1 and #2, respectively) (sFig S1b). CAF09b and CAF04 elicited similar serum IgG and IgA responses (sFig S1c). Similarly, when comparing CAF09b to CAF01 (containing DDA and the MINCLE agonist TDB), both given s.c./i.n., CAF09b elicited significantly higher CD8 T cell responses in lungs (p < 0.01, Unpaired t test) and spleen (p < 0.05, Unpaired t test) (sFig S1e), although both formulations elicited IgG and IgA responses (sFig S1f). Overall, whereas s.c./s.c. immunisation with spike formulated in CAF09b elicited only low CD8 T cell responses, which were mainly found systemically, and no IgA in the respiratory tract, s.c./i.n. or i.n./i.n. immunisation effectively generated both anti-spike IgA and respiratory tract CD8 T cell responses, the latter being partially dependent on Poly(I:C). The practical advantage of using i.n. immunisation for both priming and boosting, led us to further investigate this regimen.

### Intranasal CAF09b-adjuvanted protein subunit vaccine elicits higher upper respiratory tract immune responses than the licenced mRNA-1273 vaccine

**Fig. 2:**
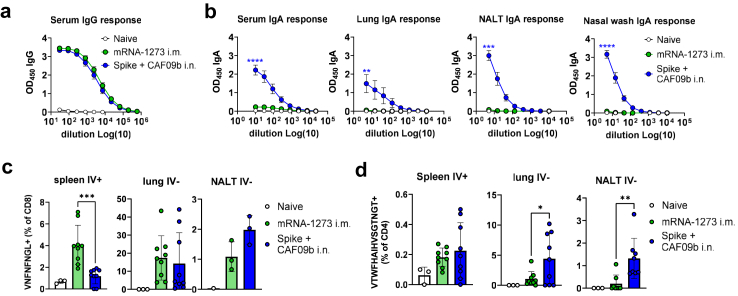
**Intranasal administration of CAF09b adjuvanted SARS-CoV-2 spike protein elicits higher respiratory tract immune responses than the licenced vaccine mRNA-1273**. Mice were immunised with two doses of SARS-CoV-2 spike HexaPro trimer (5 μg of protein) formulated in CAF09b adjuvant given intranasally (i.n./i.n.) or the licenced vaccine mRNA-1273 (Spikevax) vaccine (1 μg of mRNA given intramuscularly (i.m/i.m.)). **a)** Serum IgG antibody responses against spike protein. **b)** IgA antibody responses against spike protein measured in serum, lungs, nasal-associated lymphoid tissue (NALT), and nasal washes. Mice were injected i.v. with anti-CD45.2 to distinguish between circulating CD45+ (IV+) and tissue resident CD45− (IV−) cells **c)** CD8 T cell responses, as identified by gating on live+CD8+CD44+ cells binding a spike-specific tetramer (VNFNFNGL), were measured by flow cytometry. CD8 T cell responses were assessed systemically (IV+) in spleen (left panel), and locally (IV−) in lungs (middle panel) and NALT (right panel). Data represent two independent experiments with n = 3 (naïve) or 8–9 (vaccinated) mice per group. To obtain enough cells for analysis of CD8 T cell responses in NALT, each data point displays a pool of three mice. **d)** CD4 T cell responses, measured by gating for spike tetramer S62-76 (VTWFHAIHVSGTNGT) on live+CD8−CD19−CD4+CD62L−CD44+ cells, was assessed systemically (IV+) in spleen (left panel), and locally (IV−) in lungs (middle panel) and NALT (right panel). The experiment was performed once and data represent n = 3 (naïve) or n = 8–9 (vaccinated) mice per group. Mean ± SEM is displayed. Statistically significant differences are indicated by ∗, ∗∗, ∗∗∗ or ∗∗∗∗ (Student t-test, p < 0.05, 0.01, 0.001 or 0.0001, respectively). There were no statistically significant differences among groups unless indicated.

The mRNA-LNP vaccine mRNA-1273 (Spikevax) was successfully used during the COVID-19 pandemic and we therefore included mRNA-1273 as a clinically relevant comparison vaccine. The mRNA-1273 vaccine was given in a two-dose i.m. regimen of 1 μg of mRNA, similar to other preclinical studies.^36^ mRNA-1273 is known to elicit a strong systemic antibody response,^37^ and two i.n. immunisations with Spike + CAF09b elicited similar anti-spike serum IgG responses to mRNA-1273 given i.m. (Fig. 2a) but only CAF09b-adjuvanted vaccine administered i.n. elicited IgA responses, measured both systemically in serum and locally in the lungs, NALT and nasal washes (significantly higher than in the mRNA-1273 group, p < 0.01, Unpaired t test) (Fig. 2b).

Using MHC-I tetramer staining for S_539–546_ (VNFNFNGL), we found that the mRNA-1273 i.m. vaccine elicited high-magnitude systemic CD8 T cell responses in the spleen, which were significantly higher than in the CAF09b i.n. group (p < 0.001, Unpaired t test) (Fig. 2c). In contrast, in the lung IV- population, CD8 T cell responses were similar between mRNA-1273 i.m. and Spike + CAF09b i.n., and in the NALT, there was a tendency towards higher CD8 T cell responses amongst IV- cells in the CAF09b i.n. group compared to the mRNA-1273 i.m. group (not significant, p = 0.09, Unpaired t test) (Fig. 2c). To compare CD4 T cell responses after mRNA-1273 and CAF09b i.n. immunisation, we established an MHC class II tetramer spike_62–76_ (VTWFHAIHVSGTNGT).^20^ mRNA-1273 i.m. and Spike + CAF09b i.n. elicited similar systemic (IV+) CD4 T cell responses, measured in the spleen, whilst spike + CAF09b i.n. gave higher responses in IV− lung parenchymal and NALT CD4 T cells (p < 0.05 and 0.01, respectively) (Fig. 2d and sFig S2). Lung parenchymal CD4 T cell responses were also assessed by re-stimulating with the full-spike antigen and assaying IFN-γ and TNF-α by intracellular cytokine staining, which likewise demonstrated significantly higher responses in mice having received Spike + CAF09b i.n. compared to the mRNA-1273 i.m. vaccine (p < 0.01, Unpaired t test, sFig S3). Finally, to identify the type of T helper profile elicited by the two vaccines, we re-stimulated splenocytes with spike protein and determined cytokine secretion in the supernatant. Whilst both mRNA-1273 i.m. and Spike + CAF09b i.n. elicited IFN-γ and IL-2 secretion, IL-5, IL-10, and IL-13 were only detected in the mRNA-1273 i.m. group (significantly higher than in the CAF09b i.n. group, p < 0.05, Unpaired t test) (sFig S4). In contrast, IL-17 responses were only detected in the CAF09b i.n. group (p < 0.001). Thus, whereas mRNA-1273 i.m. elicited mostly systemic T cell responses with a balanced Th1/Th2 profile, CAF09b i.n. elicited a more Th1/Th17 biased response and higher-magnitude local respiratory tract antigen-specific CD4 T cells.

### Intranasal administration using CAF09b leads to antigen retention in the nasal cavity

**Fig. 3:**
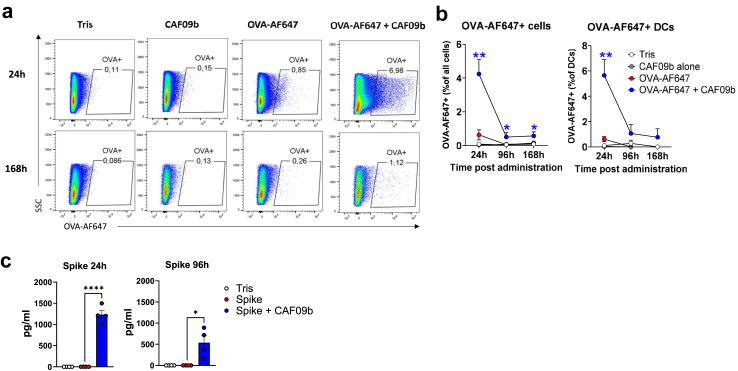
**Intranasal administration of CAF09b-adjuvanted vaccine forms a depot in the nasal cavity**. OVA-AF647 (5 μg) was administered alone or formulated in CAF09b and injected intranasally. Mice were injected i.v. with anti-CD45.2 to distinguish between circulating and tissue resident cells. Cells binding to OVA-AF647 in the nasal cavity were measured by flow cytometry at the indicated time points. Cells were gated as live+CD45-OVA+ cells. **a)** Representative plots of OVA+ cells in the nasal cavity. **b)** Total percentage of nasal cavity cells binding to OVA (left panel) or dendritic cells (gated as CD11c+MHC-II+) binding to OVA (right panel). **c)** SARS-CoV-2 spike HexaPro trimer (5 μg) was administered alone or formulated in CAF09b and injected intranasally. The concentrations of spike protein were assessed in nasal cavity homogenates after enzymatic treatment at 24 and 96 h post administration. The experiments were performed once and data represent n = 3–4 mice per group. Mean ± SEM is displayed. Statistically significant differences are indicated by ∗, ∗∗ or ∗∗∗∗ (Student t-test comparing antigen alone to antigen + CAF09b, p < 0.05, 0.01 or 0.0001, respectively).

We hypothesised that the high-magnitude local airway immune responses after i.n. administration of antigen in CAF09b may be due to the vaccine adhering to the nasal epithelium. Indeed, i.n. administration of OVA-AF647 formulated in CAF09b led to a vaccine depot in the nasal cavity, which could be measured at 24 h post i.n. administration (Fig. 3a and b). Thus, 4% of cells isolated from the nasal cavity stained positive when OVA-AF647 was given in CAF09b adjuvant, whereas less than 1% stained positive when OVA was administered alone (significant, p < 0.01, Unpaired t test). At 96 h and 168 h post administration, AF647 signal could still be detected in the nasal cavity of mice that had received OVA-AF647 formulated in CAF09b, whereas background levels were observed in mice that had received OVA-AF647 alone (significant, p < 0.05). Notably, staining for dendritic cells revealed OVA-AF647+CD11c+MHC-II+ DCs in the nasal cavity of mice that had received the CAF09b adjuvant (Fig. 3b and sFig S5). To extend these data, we used a sensitive kit based on chemiluminescence to quantify the concentrations of spike protein in the nasal cavity. Whilst spike administered without adjuvant was below detection limit, spike formulated in CAF09b was detected at both 24 and 96 h post administration at average concentrations of 1200 pg/mL and 500 pg/mL, respectively (Fig. 3c). Overall, this demonstrated that i.n. administered CAF09b retains antigen in the nasal cavity, allowing for prolonged exposure to immune cells recruited to the nasal epithelium.

### Intranasal subunit vaccine protects against SARS-CoV-2 in hACE2 mice

**Fig. 4:**
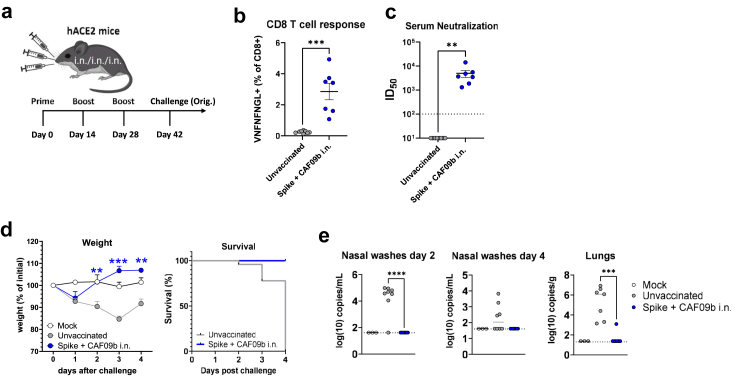
**Intranasal administration of CAF09b adjuvanted spike protein protects against SARS-CoV-2 challenge in mice**. hACE2 mice were vaccinated three times, two weeks apart, with SARS-CoV-2 spike HexaPro trimer protein formulated with CAF09b via intranasal administration. Two weeks later, the animals were challenged with 3.1 × 10^3^ TCID_50_ of the original Wu-Hu-1-like SARS-CoV-2 (SSI-H5 strain). **a)** Immunisation and challenge scheme. Immediately prior to challenge (day 40), **b)** CD8 T cell responses were evaluated by staining for tetramer (VNFNFNGL) positive cells and **c)** Serum neutralisation of SARS-CoV-2 was tested in a culture derived SARS-CoV-2 assay against the homologous Wu-Hu-1 like SARS-CoV-2/human/Denmark/DK-AHH1/2020 isolate. The dotted line indicates the limit of detection and is plotted as ID_50_ = 100. **d)** Weight change (left panel) and survival (right panel) following infection. **e)** Viral load in nasal washes at day 2 and 4 post infection and in lungs at 4 days post infection, measured by a diagnostic qPCR against the E-gene. Figures represent n = 3 (mock), n = 8 (unvaccinated) or n = 7 (vaccinated) mice per group. The latter group initially contained n = 8 mice, but one mouse was found dead five days after the 3rd immunisation (prior to challenge). Although it cannot be ruled out that this could be vaccine-related, the animal did not show any symptoms when monitored following vaccination. Statistically significant differences between unvaccinated and vaccinated animals are indicated by ∗∗, ∗∗∗ or ∗∗∗∗ (Student t-test, p < 0.01, 0.001 or 0.0001, respectively). There was no statistically significant difference among groups unless indicated. The experiment was performed once.

To test if i.n. administered CAF09b-adjuvanted spike vaccine could protect against SARS-CoV-2 infection, we initially used the highly susceptible K18-hACE2 mouse model.^38^ hACE2 mice were vaccinated three times (i.n./i.n/i.n.) with two weeks interval using CAF09b adjuvanted spike HexaPro and challenged two weeks after the last immunisation (Fig. 4a). We confirmed that vaccinated hACE2 mice had mounted spike-specific CD8 T cell responses by MHC-I tetramer staining of PBMCs prior to challenge (Fig. 4b). i.n. vaccination with spike formulated in CAF09b also elicited SARS-CoV-2 neutralising serum antibody responses against the Wu-Hu-1-like (DK-AHH1) virus (Fig. 4c). The hACE2 mice were challenged i.n. with 3.1 × 10^3^ tissue culture infective dose (TCID_50_) of homologous SARS-CoV-2 (SSI-H5 isolate). Unvaccinated control hACE2 mice rapidly lost body weight and met defined humane end-points four days after challenge (Fig. 4d). In contrast, all vaccinated animals only had transient weight loss the day following challenge and rapidly regained weight (Fig. 4d). A diagnostic qPCR for the E-gene did not detect virus in nasal washes of vaccinated mice whereas seven out of eight unvaccinated mice had detectable virus on day two post challenge and four of eight on day four (Fig. 4e). In the lungs, only one of eight mice vaccinated i.n. with CAF09b adjuvanted Spike had detectable virus (significantly lower than unvaccinated controls, p < 0.001, Unpaired t test) (Fig. 4e). Thus, three i.n. immunisations with CAF09b adjuvanted spike HexaPro vaccine effectively protected hACE2 mice against weight loss and virus infection of the upper and lower respiratory tract.

### Intranasal subunit vaccine protects against the original SARS-CoV-2 and the Omicron BA.5 variant in Syrian hamsters

**Fig. 5:**
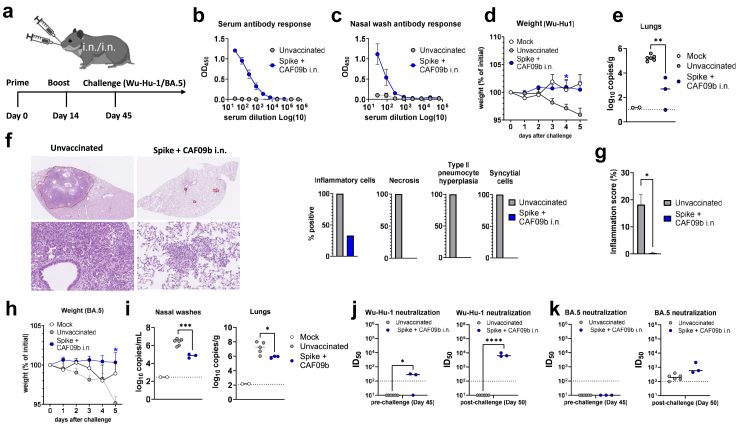
**Intranasal administration of CAF09b adjuvanted spike protein partially protects against the original SARS-CoV-2 and the Omicron BA.5 variant in hamsters**. Syrian hamsters were vaccinated twice, two weeks apart, with SARS-CoV-2spike HexaPro trimer protein (Wu-Hu-1) formulated in CAF®09b via intranasal administration. Four weeks later, the animals were challenged with 3.1 × 10^3^ TCID_50_ of either the original Wu-Hu-1-like SARS-CoV-2 (SSI-H5 strain) or the Omicron BA.5 strain. **a)** Immunisation and challenge scheme. **b)** Serum IgG antibody responses and **c)** nasal wash antibody responses (IgG/IgA/IgM) were measured against Wu-Hu-1 spike HexaPro immediately prior to challenge (Day 44). **d)** Weight loss following challenge with original Wu-Hu-1-like SARS-CoV-2 (SSI-H5 strain). **e)** Viral load in lungs at 5 days post infection, measured by a diagnostic qPCR against the E-gene. Following euthanization, the right lung lobes were pseudo-perfused fixed in 10% neutral buffered formalin and processed for histology. The H&E stained sections were examined for pulmonary pathology. **f)** In representative lung sections from unvaccinated and Spike + CAF09b i.n. immunised hamsters, the area of inflamed lung tissue has been encircled. Lower panels indicate larger magnification of encircled area. The slides were scanned using a Hamamatsu Nanozoomer S360 scanner. Plots display the frequency of hamsters in each group (n = 6 unvaccinated and n = 3 vaccinated animals) having influx of the indicated inflammatory cells, necrosis, type II pneumocyte hyperplasia and formation of syncytial cells. **g)** Lung inflammation scores were determined as the area affected by inflammation out of total lung tissue area (in percent). Bars represent group means +SEM. **h)** Weight changes following infection with the BA.5 variant. **i)** Viral load of BA.5 in nasal washes and lungs at 5 days post infection, measured by a diagnostic qPCR quantifying the E gene. Serum neutralisation against **j)** the Wu-Hu-1-like SARS-CoV-2 (DK-AHH1) strain and **k)** the BA.5 variant (DK-AHH6) was measured prior to and following challenge with BA.5. The dotted lines indicate the limit of detection and is plotted as ID_50_ = 100. Statistically significant differences between unvaccinated and vaccinated animals are indicated by ∗, ∗∗, ∗∗∗ or ∗∗∗∗ (Student t-test, p < 0.05, 0.01, 0.001 or 0.0001, respectively). There were no statistically significant differences among groups unless indicated. Figures represent n = 2 (mock), 6 (unvaccinated) or 3 (vaccinated) hamsters per group. The experiments were performed once.

Compared to hACE2 mice, the Syrian hamster model better reflects SARS-CoV-2 infection in humans.^39^ We evaluated the CAF09b adjuvanted vaccine in Syrian hamsters using a two-dose i.n./i.n. regimen (two weeks apart) followed by challenge four weeks later (Fig. 5a). CAF09b adjuvanted SARS-CoV-2 spike HexaPro elicited spike-specific IgG (Fig. 5b) in the serum. Spike-specific antibody responses could also be detected in nasal washes from the CAF09b-vaccinated group, but not in unvaccinated controls prior to challenge (Fig. 5c). Spike + CAF09b i.n./i.n. vaccination prevented weight loss (Fig. 5d) and significantly reduced virus titres in the lower respiratory tract (p < 0.05, Unpaired t test) (Fig. 5e). Thus two i.n. immunisations with CAF09b adjuvanted spike HexaPro vaccine effectively controlled SARS-CoV-2 infection in hamsters. Lung pathology following SARS-CoV-2 infection includes infiltration with mononuclear cells, predominantly macrophages, and neutrophils as well as hyperplasia of type-II pneumocytes.^28^^,^^39^^,^^40^ To test if i.n. vaccination with CAF09b-adjuvanted spike protein could protect against pulmonary pathology, lungs were examined at day 5 post infection. Unvaccinated animals had marked influx of inflammatory cells into the alveolar tissue as well as type II pneumocyte hyperplasia, syncytial cell formation and necrosis. Among the vaccinated animals, only one showed presence of inflammatory cells and no presence of, syncytial cells, type II pneumocytes or necrosis (Fig. 5f) and the area of inflamed lung tissue was significantly reduced compared to in unvaccinated animals (Fig. 5g). Thus, i.n. immunisation with CAF09b-adjuvanted spike vaccine protected against lung pathology in the hamster model, although it did not completely prevent virus infection of the lung. This was confirmed by lung immunohistochemistry showing widespread SARS-CoV-2 nucleocapsid staining in the unvaccinated group, but also a few pinpoints in the Spike + CAF09b i.n. immunised hamsters (sFig S6b).

To assess protection against a heterologous SARS-CoV-2 strain, we performed challenge studies in hamsters using the Omicron BA.5 variant. As in the study using homologous virus, the i.n. CAF09b-adjuvanted HexaPro (Wu-Hu-1 spike) vaccine was given as a two-dose regimen (two weeks apart) followed by challenge four weeks later (Fig. 5a). The vaccine protected against weight loss (statistically significant compared to unvaccinated controls, p < 0.05) (Fig. 5h). Virus titres were measured on days two and five post challenge. On day two there was no difference in virus titres between unvaccinated and vaccinated animals (data not shown), whilst at day five significantly reduced titres were observed in vaccinated animals in nasal washes (p < 0.001, Unpaired t test) and lungs (p < 0.05, Unpaired t test) (Fig. 5i). Measuring serum neutralising antibody titres, two of three animals had neutralising antibodies against the Wu-Hu-1 (DK-AHH1) strain prior to challenge. These were rapidly boosted in all vaccinated animals after challenge with BA.5, but not in unvaccinated controls (significantly different from unvaccinated controls, p < 0.0001, Unpaired t test) (Fig. 5j). In contrast, no neutralising antibodies against BA.5 were measured in the vaccinated group prior to challenge and these were of the same magnitude as in unvaccinated controls after challenge (Fig. 5k). Thus, i.n. immunisation with CAF09b-adjuvanted vaccine cross-protected against weight loss and significantly lowered virus load upon challenge with a heterologous SARS-CoV-2 strain, despite low-level systemic neutralising antibody responses.

## Discussion

Intranasal vaccines are attractive due to ease of administration and ability to protect against infection of the upper airways. Here we report on an intranasal vaccine strategy applying a cationic liposomal adjuvant stimulating the C-type lectin receptor MINCLE as well as TLR3. As proof-of-concept, we tested this in combination with recombinant prefusion-stabilised SARS-CoV-2 spike HexaPro protein in mice and found that the adjuvant stimulated NALT and lung CD8 T cell responses. Furthermore, it elicited IgA responses in the upper and lower airways. Importantly, these responses were not observed when the same vaccine, or the licenced mRNA vaccine mRNA-1273, was given parenterally.

None of the vaccines available during the first waves of the SARS-CoV-2 pandemic were developed for airway administration. The first licenced vaccines, mRNA encapsulated in lipid nanoparticles and administered intramuscularly, were highly effective at protecting against severe disease but had limited effect against virus transmission.^41^^,^^42^ A few intranasally administered vaccines were then developed,^6^^,^^43^ but when the first intranasal vaccines were approved the pandemic had already reached a stage where it was difficult to determine their benefit against primary infection. Although there is still limited experience with i.n. SARS-CoV-2 vaccines in the clinic, there is substantial evidence from pre-clinical studies that i.n. administered vaccines may be superior to parenterally administered vaccines for eliciting upper respiratory tract immunity protecting against virus transmission. Thus, the live-attenuated virus vaccine candidate sCPD9 induced rapid viral clearance in Syrian hamsters and protected against SARS-CoV-2 transmission as opposed to the mRNA vaccine BNT162b2.^44^^,^^45^ Previous studies using parenteral mRNA prime followed by mucosal boosting also demonstrated strong CD8 T cell responses and IgA in the upper airways.^20^ We previously described a parenteral prime – i.n. booster vaccine using the CAF01 adjuvant, which elicited upper respiratory tract IgA and protected against onward transmission.^28^ From a practical perspective, a more optimal vaccine regimen would be i.n. immunisation for both priming and boosting. The i.n.-only administered vaccines also have the advantage of being given needle-free, which eliminates risks associated with injectables, such as transmission of blood-borne diseases. In the present study we tested the CAF09b adjuvanted SARS-CoV-2 spike HexaPro vaccine using an i.n. only regimen. We noticed that the vaccine retained antigen in the nasal cavity for prolonged stimulation, an effect which is probably attributed to the DDA component forming electrostatic interactions with epithelial proteins. The i.n. vaccine effectively induced respiratory tract IgA, CD4 and CD8 T cell responses, the latter being partially dependent on Poly(I:C), and protected against SARS-CoV-2 challenge with the homologous Wu-Hu-1 strain in hACE2 mice. In Syrian hamsters the vaccine protected against disease and lung pathology, but did not fully clear the virus. This difference between mice and hamsters could be due to species differences, but could also be because of different vaccine regimens, as hACE2 mice received three immunisations two weeks apart and were challenged two weeks after the last immunisation, whilst Syrian hamsters only received two immunisations and were challenged four weeks after the last immunisation. In Syrian hamsters the vaccine protected against weight loss and significantly reduced upper respiratory tract and lung virus titres following challenge with the Omicron BA.5 strain and this was observed despite the lack of detectable serum BA.5 neutralising antibody responses, which may suggest a role for local or systemic T cell responses for the protection.^14^^,^^46^ A limitation of the studies was that we did not determine the mechanism of protection against the heterologous strain. Also, we did not directly determine if the i.n. administered vaccine protected against virus transmission.

The SARS-CoV-2 pandemic rapidly spread throughout the world and caused high mortality. Development of improved vaccination strategies effectively protecting against both disease and virus transmission is important to prepare for future pandemics caused by respiratory viruses. Here we describe a novel intranasal protein-based vaccine effectively protecting against SARS-CoV-2 in mouse and hamster models as a proof-of concept for intranasally delivered vaccines against respiratory viruses.

## Contributors

K.W., S.T.S., J.Z., A.T., C.P., K.T.H., K.I., L.K.I., G.E., G.J., I.R., A.O., J.G., K.H., H.E.J., S.R., F.F., J.B., and G.K.P. designed research. K.W., S.T.S., J.Z., A.T., C.P., C.F.A., K.T.H., R.F.J., and J.S.H. performed experiments. K.W., J.Z., S.T.S., C.F.A., K.T.H., R.F.J., J.S.H., H.E.J., S.R., and G.K.P. analysed data. K.W., S.T.S., J.B., and G.K.P. wrote the paper. All authors read and approved the final version of the manuscript and have had access to the raw data. K.W., S.T.S., and G.K.P. can verify the accuracy of the raw data for the study.

## Data sharing statement

Study protocol and all data collected for the study, including raw data and data analysis will be made available to others upon request. All data will be available upon publication of the manuscript, by contacting the corresponding author. Data will be made available after approval of a proposal and with a signed data access agreement.

## Declaration of interests

The authors declare that there are no competing interests.

## Appendix ASupplementary data

Figs S1–S6

Captions
