# Supplementary figures and images for: Intranasal recombinant protein subunit vaccine targeting TLR3 induces respiratory tract IgA and CD8 T cell responses and protects against respiratory virus infection
Source: eBioMedicine. 2025 Feb 20;113:105615. doi: 10.1016/j.ebiom.2025.105615 (PMC11893338; doi:10.1016/j.ebiom.2025.105615)

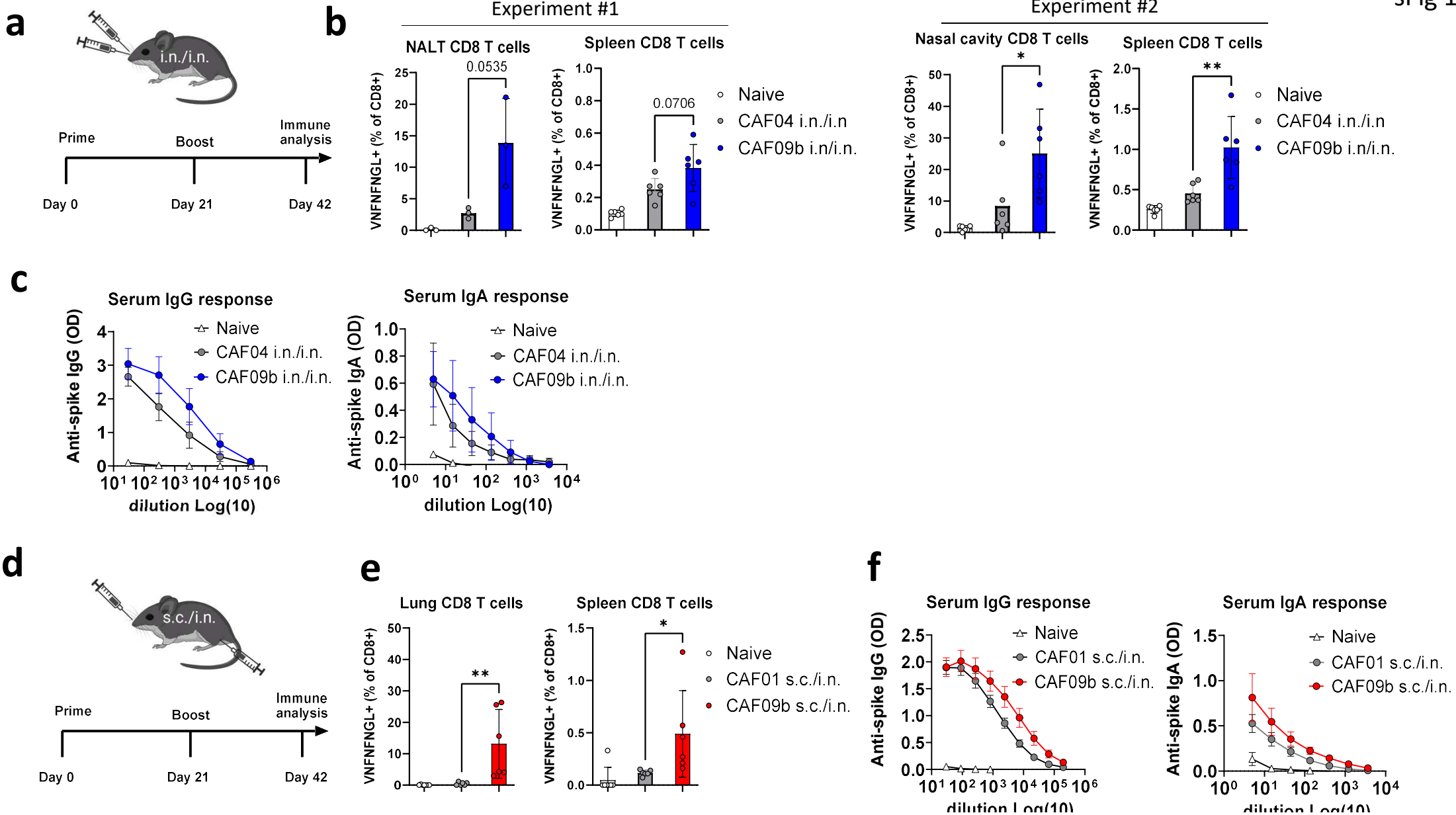

a

mRNA-1273

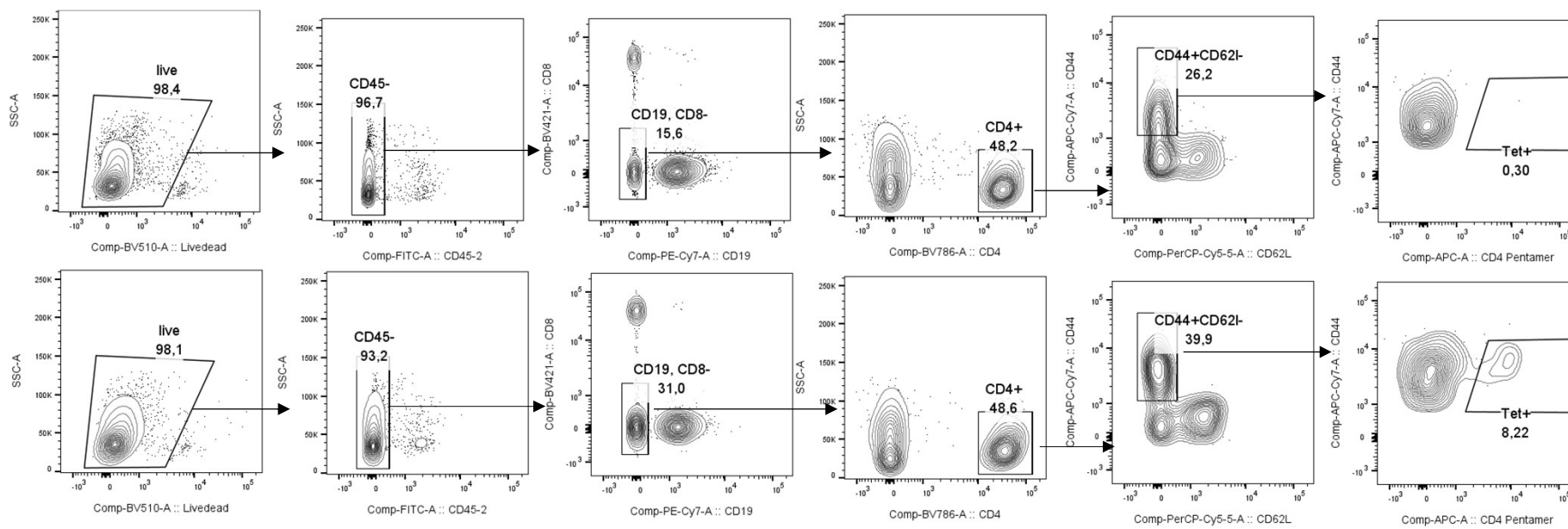Spike+  
CAF09b i.n.

b

mRNA-1273

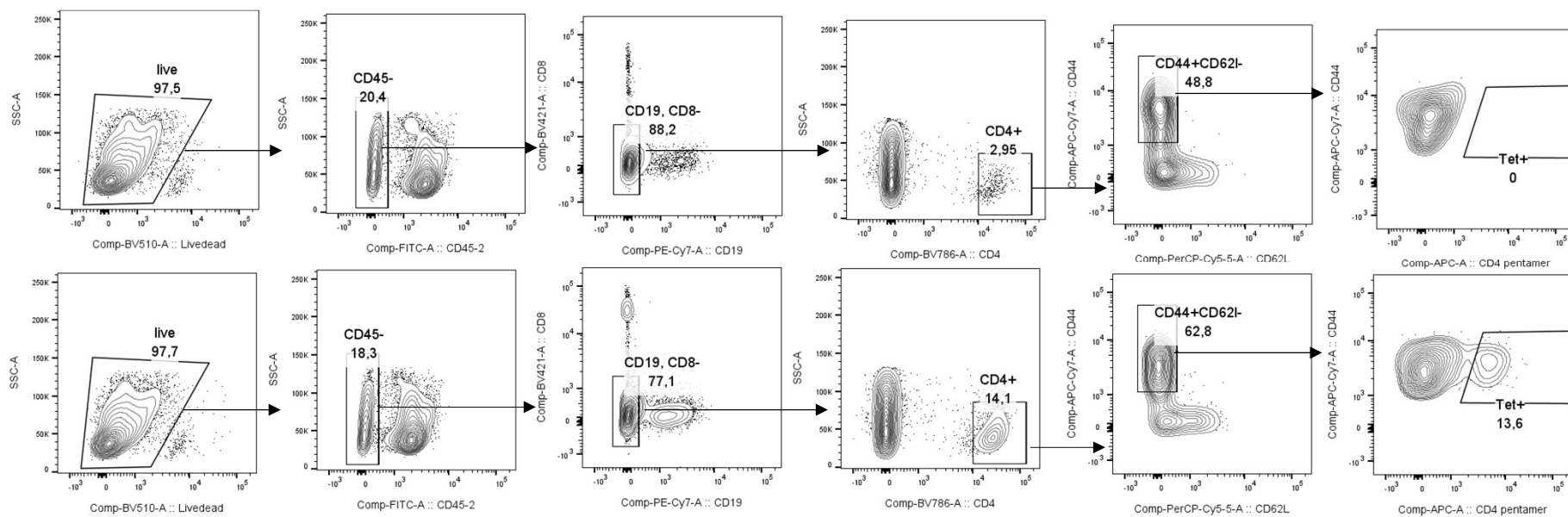Spike+  
CAF09b i.n.

sFig 3

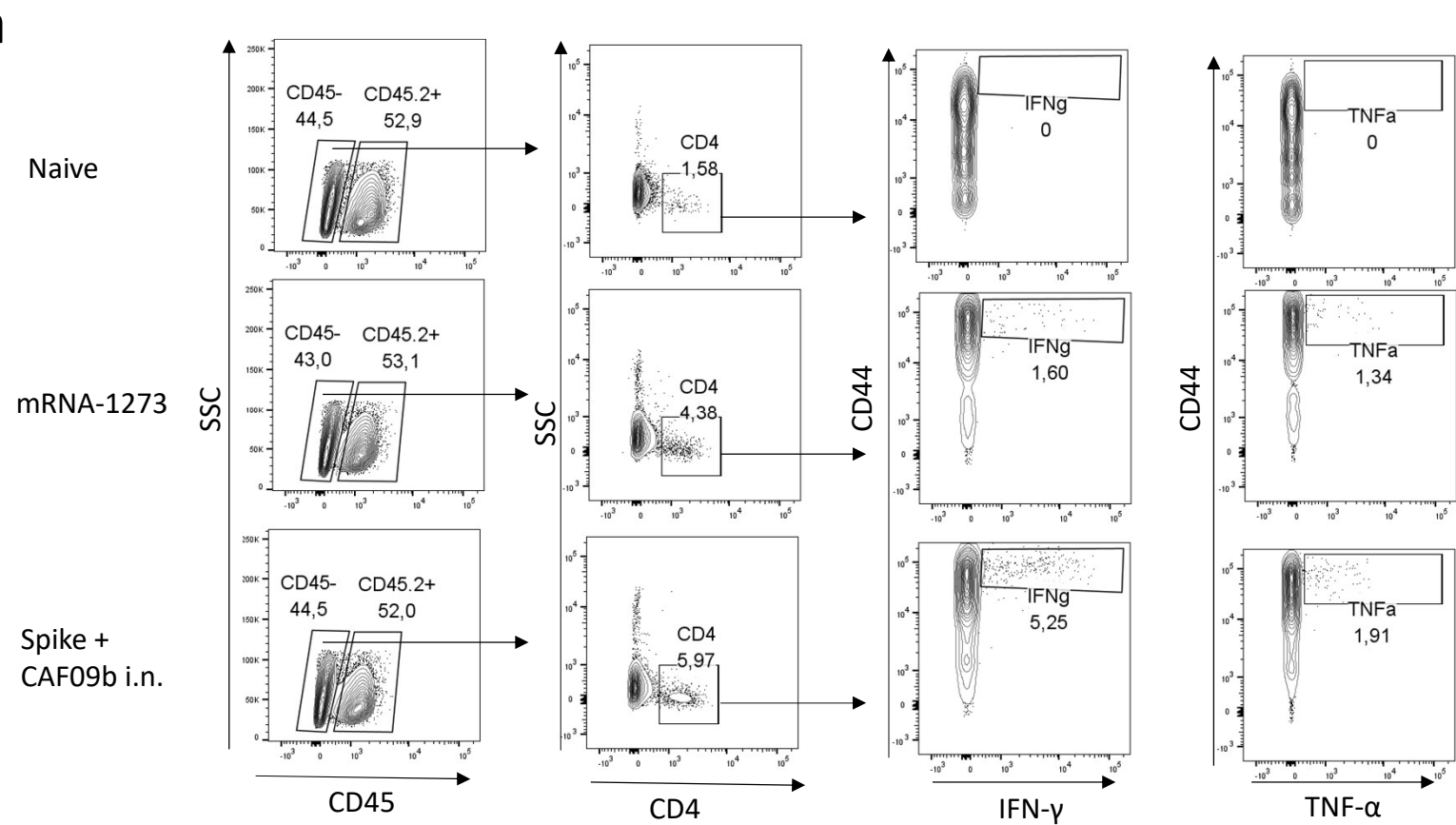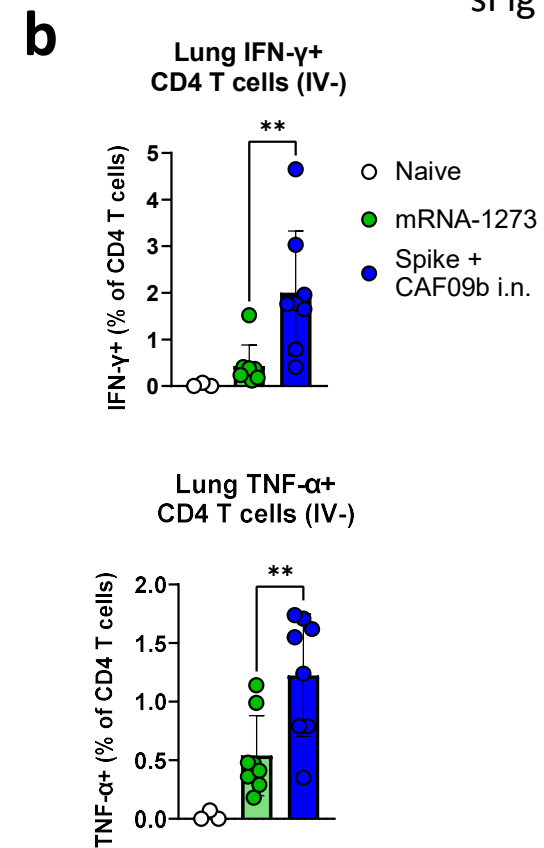

sFig 4

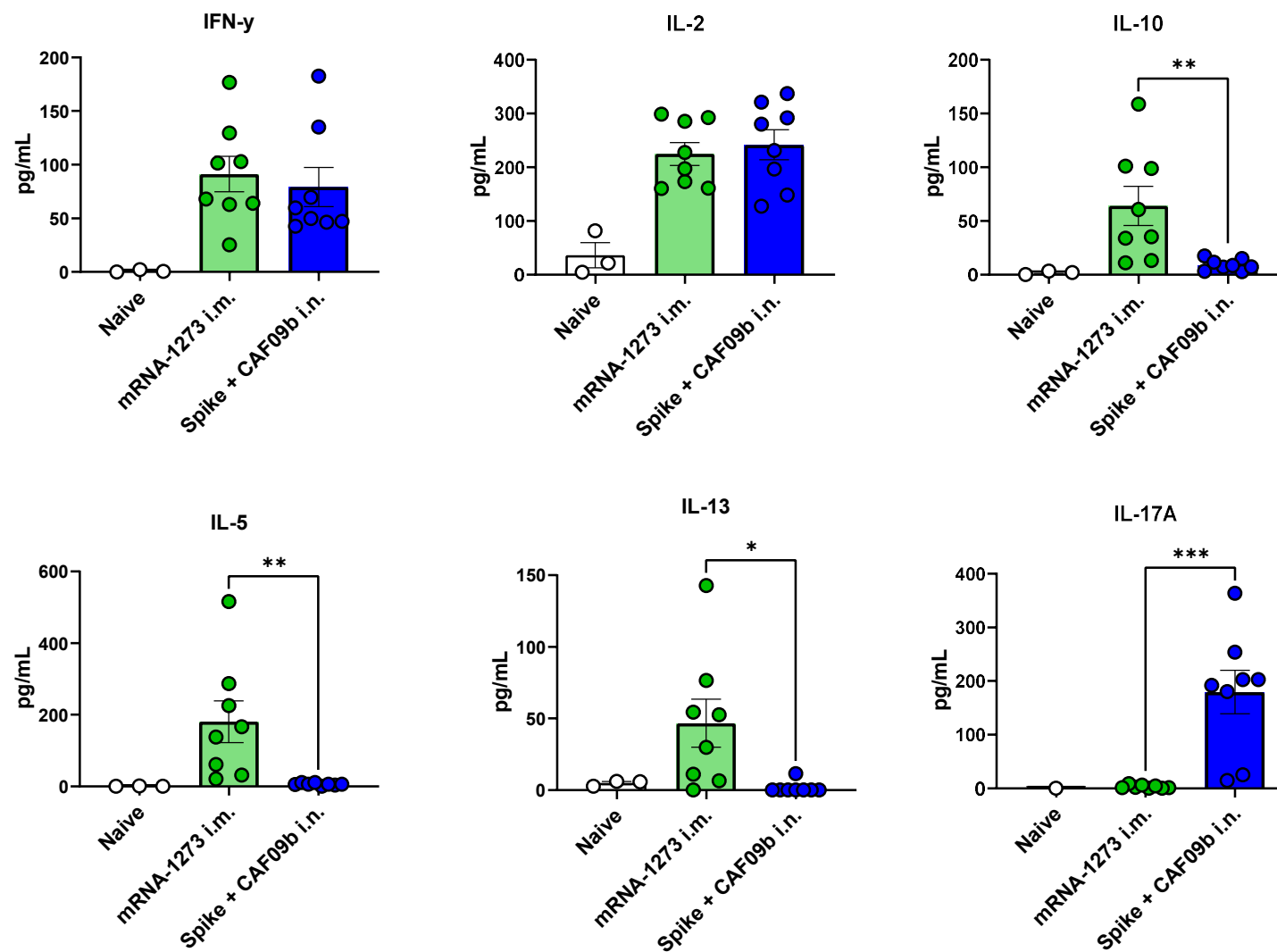

sFig 5

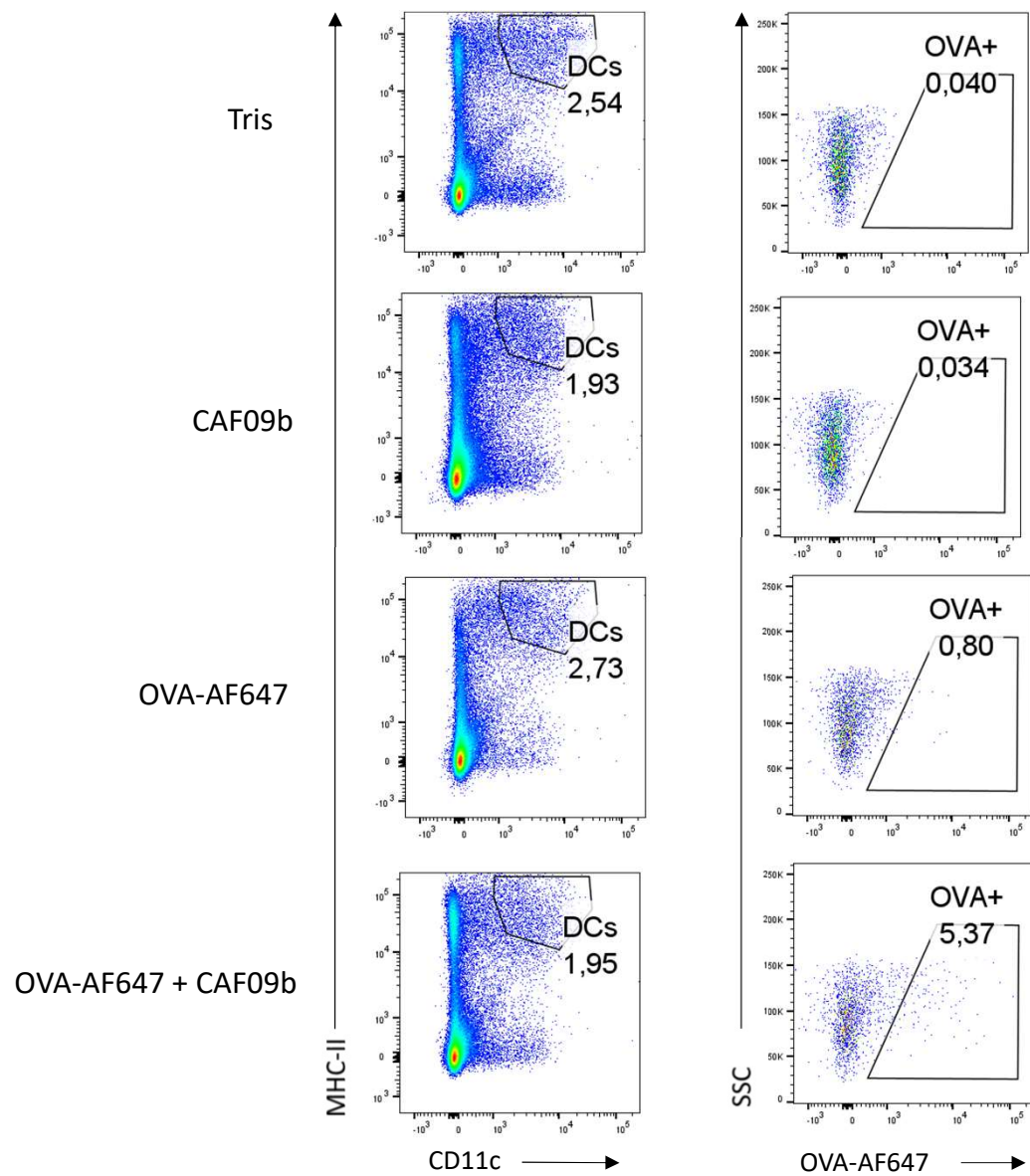

**a****Unvaccinated**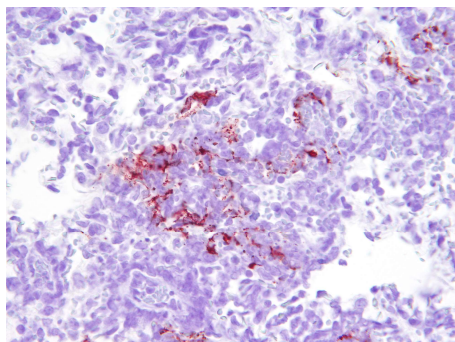**Spike + CAF09b i.n.**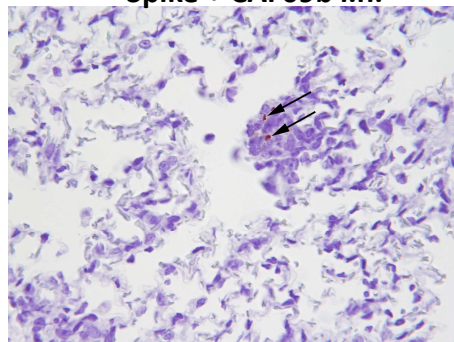

Supplement: Figs S1–S6 [file mmc1.pdf]
