# Supplementary material for: Intranasal recombinant protein subunit vaccine targeting TLR3 induces respiratory tract IgA and CD8 T cell responses and protects against respiratory virus infection
Source: eBioMedicine. 2025 Feb 20;113:105615. doi: 10.1016/j.ebiom.2025.105615 (PMC11893338; doi:10.1016/j.ebiom.2025.105615)
Supplement: Captions [file mmc2.docx]

**Caption for supplementary material**

**sFig 1 CAF09b-induced CD8 T cell responses in the airways after intranasal immunization are partially dependent on the TLR3 agonist Poly (I:C)**

**a)** Mice were immunized twice with SARS-CoV-2 spike HexaPro trimer protein formulated in cationic liposomes containing DDA formulated with the MINCLE agonist MMG (CAF^®^04) or DDA/MMG in combination with Poly (I:C) (CAF^®^09b). The vaccine was administered as two intranasal immunizations (i.n./i.n.). Mice were injected i.v. with anti-CD45.2 to distinguish between circulating and tissue resident cells. **b)** The frequency of live CD8+CD44+ cells binding a spike-specific tetramer (VNFNFNGL) was determined by flow cytometry in nasal associated lymphoid tissue (NALT, left panel) and spleen (right panel) in experiment #1 and in nasal cavity (left panel) and spleen (right panel) in experiment #2. Data represent n = 6 mice per group in each experiment. For NALT, each data point represents two pooled samples. **c)** Serum spike-specific IgG (left panel) and IgA (right panel)**.** Data represent n = 6 mice per group. The experiment was performed once. **d)** Mice were immunized twice with SARS-CoV-2 spike HexaPro trimer protein formulated in cationic liposomes containing DDA formulated with the MINCLE agonist TDB (CAF^®^01) or DDA/MMG in combination with Poly (I:C) (CAF^®^09b). The vaccine was administered as subcutaneous prime - intranasal boost (s.c./i.n.). **e)** The frequency of live CD19-CD4-CD8+CD44+ cells binding a spike-specific tetramer (VNFNFNGL) was determined by flow cytometry in lungs (left panel) and spleen (right panel). **f)** Serum spike-specific IgG (left panel) and IgA (right panel)**.** Data represent n = 6 mice per group. The experiment was performed once. Mean ± SEM is displayed. Statistically significant differences are indicated by * or ** (Student t-test, p<0.05 or 0.01, respectively).

**sFig 2 Representative plots of airway antigen-specific CD4 T cells**

Mice were immunized with two doses of SARS-CoV-2 spike HexaPro trimer (5μg of protein) formulated in CAF09b adjuvant given intranasally (i.n./i.n.) or the licensed vaccine mRNA-1273 (Spikevax) (1 μg of mRNA given intramuscularly (i.m/i.m.)). Mice were injected i.v. with anti-CD45.2 to distinguish between circulating CD45+ (IV+) and tissue resident CD45- (IV-) cells. Representative stainings of CD4 T cell responses, measured by gating for spike tetramer S62-76 (VTWFHAIHVSGTNGT) on live+CD8-CD19-CD4+CD62L-CD44+ cells, assessed in (IV-). CD19 and CD62L were used to gate out B cells and naïve T cells, respectively. **a)** NALT and **b)** Lungs. The experiment was performed once.

**sFig 3 Intranasal CAF09b adjuvanted spike subunit vaccine is superior to mRNA-1273 for eliciting lung CD4 T cell responses**

Mice were immunized with two doses of SARS-CoV-2 spike HexaPro trimer (5μg of protein) formulated in CAF09b adjuvant given intranasally (i.n./i.n.) or the licensed vaccine mRNA-1273 (Spikevax) (1 μg of mRNA given intramuscularly (i.m/i.m.)). Mice were injected i.v. with anti-CD45.2 to distinguish between circulating CD45+ (IV+) and tissue resident CD45- (IV-) cells. At the day of termination, lungs were processed and restimulated with full spike protein. Intracellular cytokines (IFN-γ and TNF-α), measured by gating on parenchymal (IV-) CD4+CD44+ cells, were then measured by flow cytometry. **a)** Representative plots of IFN-γ and TNF-α producing CD4 T cells in the lungs. **b)** Frequency of cells producing the individual cytokines. The experiment was performed once and data represent n = 3 (naïve) or 8 (vaccinated) mice per group. Statistically significant differences are indicated by ** (Student t-test, p< 0.01).

**sFig 4 Intranasal CAF09b adjuvanted spike subunit vaccine elicits systemic Th1/Th17 responses**

Mice were immunized with two doses of SARS-CoV-2 spike HexaPro trimer (5μg of protein) formulated in CAF09b adjuvant given intranasally (i.n./i.n.) or the licensed vaccine mRNA-1273 (Spikevax) (1 μg of mRNA given intramuscularly (i.m/i.m.)). At the day of termination, splenocytes were re-stimulated with HexaPro spike protein and secreted cytokines were measured in the supernatant. Bars show mean +/- SEM. Statistically significant differences are indicated by *, ** or *** (Student t-test, p<0.05, 0.01, 0.001, respectively). The experiment was performed once.

**sFig 5 Representative stainings of OVA-binding dendritic cells in the nasal cavity**

OVA-AF647 (5μg) was formulated in CAF09b and administered intranasally. Mice were injected i.v. with anti-CD45.2 to distinguish between circulating (IV+) and tissue resident (IV-) cells. IV- Cells binding to OVA-AF647 in the nasal cavity were measured by flow cytometry. Cells were gated as live+CD45-OVA+ cells. Representative plots of CD11c+MHC-II+ cells binding to OVA at 24 h post injection are displayed. The experiment was performed once.

**sFig6 Intranasal administration of CAF09b adjuvanted spike protein reduces lung inflammation**

Syrian hamsters were vaccinated twice, two weeks apart, with SARS-CoV-2 spike HexaPro trimer protein (Wu-Hu-1) formulated in CAF®09b via intranasal administration. Four weeks later, the animals were challenged with 3.1 x 10^3^ TCID_50_ of the original Wu-Hu-1-like SARS-CoV-2 (SSI-H5 strain). Following euthanization, the right lung lobes were pseudo-perfused fixed in 10% neutral buffered formalin and processed for histology, and H&E stained sections were examined for pulmonary lesions. **a)** Immunohistochemistry for SARS-CoV-2 nucleocapsid showed widespread staining in the unvaccinated animals, but only a few positive pinpoints (arrows) in the Spike + CAF09b i.n. immunized hamsters.
